# Conditional *Wwox* Deletion in Mouse Mammary Gland by Means of Two Cre Recombinase Approaches

**DOI:** 10.1371/journal.pone.0036618

**Published:** 2012-05-04

**Authors:** Brent W. Ferguson, Xinsheng Gao, Hyunsuk Kil, Jaeho Lee, Fernando Benavides, Martin C. Abba, C. Marcelo Aldaz

**Affiliations:** 1 Department of Molecular Carcinogenesis, The University of Texas M.D. Anderson Cancer Center, Smithville, Texas, United States of America; 2 CINIBA, Facultad de Medicina, Universidad Nacional de La Plata, La Plata, Argentina; Sanford Burnham Medical Research Institute, United States of America

## Abstract

Loss of *WWOX* expression has been reported in many different cancers including breast cancer. Elucidating the function of this gene in adult tissues has not been possible with full *Wwox* knockout models. Here we characterize the first conditional models of *Wwox* ablation in mouse mammary epithelium utilizing two transgenic lines expressing Cre recombinase, *keratin 5-Cre* (*BK5-Cre*) and *MMTV-Cre*. In the *BK5-Cre* model we observed very efficient *Wwox* ablation in *KO* mammary glands. However, *BK5-Cre Wwox KO* animals die prematurely for unknown reasons. In the *MMTV-Cre* model we observed significant ablation of *Wwox* in mammary epithelium with no effect on survival. In both of these models we found that *Wwox* deletion resulted in impaired mammary branching morphogenesis. We demonstrate that loss of *Wwox* is not carcinogenic in our *KO* models. Furthermore, no evidence of increase proliferation or development of premalignant lesions was observed. In none of the models did loss of a single *Wwox* allele (i.e. haploinsufficiency) have any observable phenotypic effect in mammary gland. To better understand the function of Wwox in the mammary gland, transcriptome profiling was performed. We observed that *Wwox* ablation results in the deregulation of genes involved in various cellular processes. We found that expression of the non-canonical Wnt ligand, *Wnt5a*, was significantly upregulated in *Wwox KO* mammary epithelium. Interestingly, we also determined that components of the Jak/Stat3 signaling pathway were upregulated in *KO* mice and this correlated with a very robust increase in phospho-Stat3 signaling, which warrants further testing. Even though the loss of Wwox expression in breast and other cancers is very well documented, our findings suggest that Wwox does not act as a classical tumor suppressor as previously thought.

## Introduction

The WW domain-containing oxidoreductase *(WWOX)* gene was identified as a potential tumor suppressor gene mapping to chromosomal region 16q23 [1], [2]. The *WWOX* gene spans >1.1 Mb and overlaps the *FRA16D* common chromosomal fragile site [1], [2], [3]. Disruptions of the *WWOX* locus by hemi, homozygous deletions and other rearrangements have been reported in various human malignancies including breast, prostate, ovarian, lung, liver cancer and multiple myeloma [1], [4], [5]. *WWOX* is highly conserved throughout evolution and in mouse the *Wwox* gene is 93% identical to its human counterpart. Interestingly the mouse *Wwox* gene also co-localizes with the murine chromosomal fragile site *Fra8E1* [6].

Significant research has been performed to elucidate the normal and putative tumor-suppressive roles of *WWOX*. The WWOX protein has a molecular mass of 46 kD with two functional motifs consisting of two N-terminal WW domains and a short-chain dehydrogenase (SDR) domain [1], [7]. The WW domains of *WWOX* have been found to be essential for protein-protein interactions with proteins harboring PPXY motifs [8] and the substrate/s for the *WWOX* NADH/NADPH oxidoreductase enzymatic domain are yet to be identified. Several potential protein partners have been identified and include regulators of cell growth and apoptosis as well as various transcription factors [8], [9], [10], [11], [12], [13]. The putative role of *WWOX* as a tumor suppressor has been suggested by various studies observing suppressed *in vivo* tumorigenicity of human cancer cells when *WWOX* was ectopically overexpressed [14], [15], [16], [17].

Our group, as well as others, have generated mouse models of *Wwox* knockdown to gain a better understanding of the *in vivo* functions of *Wwox* in development and cancer. Aqeilan et al. generated a full knockout (KO) mouse model *via* targeted disruption of the gene. Even though all mice die by 3–4 weeks these authors reported the development of periostal osteosarcomas but only in 3 of 11 pups analyzed [18]. Our group generated and characterized mice harboring a conditional *Wwox* allele in which exon 1 was flanked by loxP sites [19]. Upon breeding these mice with *EIIA-Cre* transgenic mice, we generated full *Wwox KO* mice and observed that these mice displayed severe metabolic defects, growth retardation and all mice died by 3 weeks of age as previously observed [19], however in contrast with the findings of Aqeilan and coworkers no evidence of spontaneous neoplasia was detected in these short-lived *Wwox KO* mice [19].

We are interested in understanding the normal and putative tumor-suppressive functions of *WWOX* in more detail. Since we and others observed consistent and significant loss of *WWOX* expression in breast cancer [20], [21], [22], [23] we focused first in the mammary gland. Here we report the characterization of conditional ablation of *Wwox* in mouse mammary gland using two approaches, we utilized transgenic lines with *Cre* recombinase under control of a *Keratin 5* promoter *(BK5-Cre)* and also under the *MMTV* promoter *(MMTV-Cre)* in order to inactive *Wwox* in the mammary epithelium.

## Results

### Assessment of two *Wwox* conditional KO models in mouse mammary epithelium

To determine the role of Wwox in the murine mammary gland, *Wwox ^flox/flox^* mice were mated with mice carrying a transgene expressing Cre recombinase resulting in the conditional deletion of the *Wwox* gene in mammary epithelium. To this end we tested two independent models of *Cre* driven recombination. The first conditional KO model had *Cre* recombinase expression driven by the bovine keratin 5 promoter *(BK5-Cre)*. The *BK5* promoter is activated *in utero* (E13.5) in epithelial precursor cells [24]. In the second model, the *MMTV* promoter drove *Cre* expression *(MMTV-Cre).* The *MMTV* promoter in the transgenic Line D is expressed in various tissues and is maximally turned on in mammary gland epithelium at approximately 22 days of age [25].

**Figure 1:**
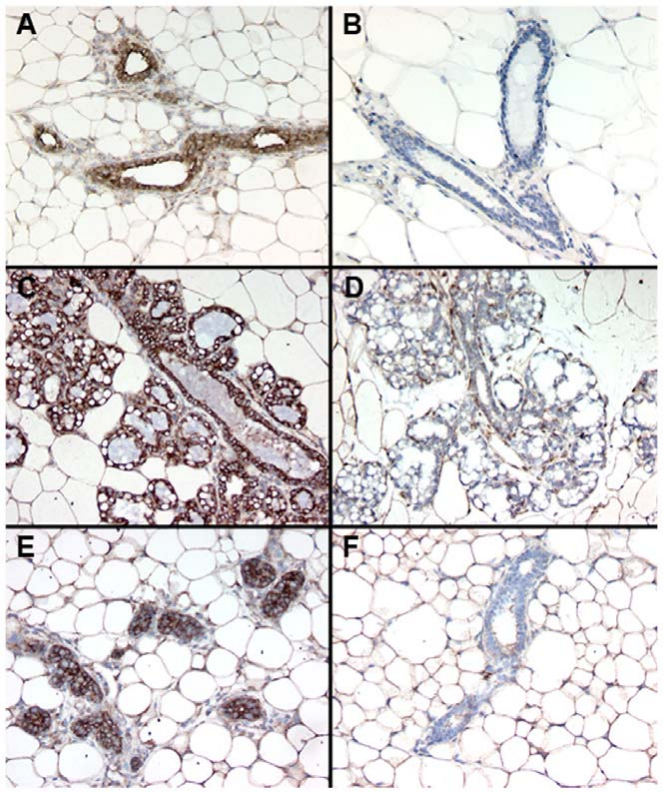
Conditional *KO* of *Wwox via BK5-Cre* and *MMTV-Cre*. (A–F) Immunostaining of formalin-fixed histological sections of mammary glands from each model of deletion reveals strong cytoplasmic Wwox staining in *WT* samples and significant loss of Wwox protein expression in *KO* animals. (A) 8 week virgin *BK5 Wwox WT*, (B) 8 week virgin *BK5 Wwox KO*, (C) P18.5 days *BK5 Wwox WT*, (D) P18.5 days *BK5 Wwox KO*, (E) 24 week virgin *MMTV Wwox WT*, (F) 24 week *MMTV Wwox KO*. Rabbit anti-Wwox [14] antibody was used at a 1∶50 dilution and counterstained with hematoxylin. All images were obtained at the same 10× magnification.

To examine Wwox protein expression and the efficacy of Wwox ablation in each of these models we analyzed histological sections of mammary glands from female mice stained with Wwox antibody. We observed high levels of Wwox expression in the cytoplasm of mammary epithelial cells of 8 week virgin *BK5 Wwox WT* control mice and this expression was largely absent in *BK5 KO* mice (Figures 1 A and B). This loss of Wwox expression was also clear in mammary glands from pregnant (P18.5 days) *Wwox KO* mice even after the burst of epithelial proliferation associated with pregnancy (Figures 1 C and D). In the *MMTV-Cre* model we found that Wwox ablation was very effective as well (Figures 1 E and F). We observed however isolated patches of epithelial structures expressing Wwox indicative of some mosaicism but only in some mice. This heterogeneity in the *MMTV Cre* model is not uncommon with Cre systems and is likely due to the mosaic nature of *Cre* expression and perhaps incomplete recombination affecting some cells in some mice. However, this mosaicism does not result in repopulation of the mammary gland by Wwox-positive epithelium as discussed in a follow up section.

**Figure 2:**
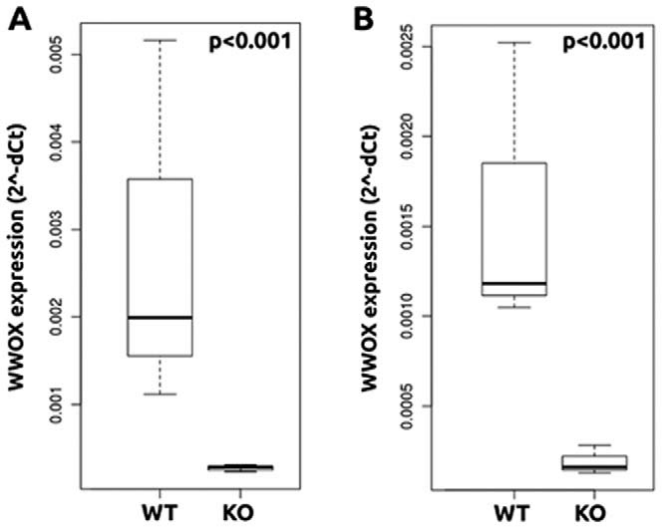
*Wwox* mRNA expression in *BK5-Cre* model of Wwox deletion. qRT-PCR was used to determine the mRNA expression of *Wwox* in mammary epithelial organoids from *BK5 Wwox WT* and *KO* animals. The box plots show dramatically decreased *Wwox* mRNA levels in epithelium from 8 week virgin (A) and P18.5 days (B) *Wwox KO* mammary epithelium when compared to their respective *WT* counterparts. Mammary epithelial organoids were isolated from 3 different *WT* and 3 different *KO* mice in each group. Samples from each mouse were run individually in triplicate to assess *Wwox* expression. Samples were normalized to 18S expression.

We further analyzed the extent of *Wwox* deletion in the *BK5-Cre* model at the RNA level by real-time PCR. We observed that *Wwox* expression was dramatically decreased in both 8 week virgin and P18.5 *Wwox KO* mammary epithelial organoids when compared to their *WT* counterparts (Figure 2). These results demonstrate that *Wwox* deletion by use of the *BK5-Cre* and *MMTV-Cre* transgenic lines is very effective in mouse mammary epithelium.

### Long-term follow up experiments *BK5-Cre* model

In order to determine the effects of long-term *Wwox* ablation in the mammary gland and to test whether lack of *Wwox* expression was tumorigenic we set up standard survival experiments. *BK5-Cre^+^; Wwox ^flox/flox^* males and females survived weaning, were fertile and females were able to lactate. However, inexplicably, *BK5 KO* mice died prematurely at an early age. The youngest animal died at 68 days of age and no *BK5 KO* mouse survived longer that 117 days of age (0% survival, n = 22). This premature death was independent of gender as 9 females and 13 males were included in the study. *BK5-Cre^(−)^; Wwox ^flox/flox^ (WT),* mice exhibited 100% survival over the same observation period. It is also important to note that loss of one *Wwox* allele in the *BK5-Cre^(+)^; Wwox^+/flox^* heterozygous mice had no effect on survival (100% survival, n = 41, Figure S1). Numerous groups of experimental animals were generated independent of the described survival study with the same results. We performed comprehensive histopathological, hematological and blood chemistry analyses on these *BK5-Cre Wwox KO* mice before dead and could not determine a reason for the relatively sudden deaths.

We observed no tumor formation in any of the *BK5 KO* mammary glands during their relatively short lifespan (mean = 115 days). Importantly, none of the *BK5-Cre^(+)^; Wwox^+/flox^* heterozygous mice develop tumors even after over a year of follow up, indicating that lack of one *Wwox* allele (i.e. equivalent to haploinsufficiency) is not tumorigenic in the mouse mammary gland.

Since *Keratin 5* is highly expressed in cells of the basal layer of multiple epithelia including the skin, we carefully inspected the epidermis of *BK5 KO* mice. We confirmed lack of Wwox expression in epidermis of *BK5 KO* mice by means of immunohistochemistry (data not shown). We observed no obvious macro or microscopic abnormal skin phenotype. Furthermore, *BK5 KO* mice displayed the normal bilayer architecture of mouse skin with no signs of hyperplasia or abnormalities in differentiation. No skin premalignant or tumorigenic lesions spontaneously developed in either *BK5 KO* or heterozygous mice.

Histopathological evaluation of mammary gland tissue sections from multiple *BK5 KO* and *Wwox^+/flox^* mice at various stages of development, revealed no signs of malignancy or premalignant changes. As can be observed in Figure 1 B no evidence of hyperplasia is noted. Similarly, no evidence of malignancy or hyperplastic nodules were observed in any of the whole mount preparations obtained from *BK5 KO* mice.

Keratin 5 immunostaining on *BK5 KO* mammary gland revealed no obvious abnormalities in differentiation (Figure S2). We also analyzed the effect of *Wwox* ablation on proliferation in the mammary gland *via* immunohistochemical staining for Ki-67 and found no statistical difference between *WT* and *KO* mice in the *BK5-Cre* model (data not shown). Importantly, we also found no difference in ERα or PR expression between the *BK5 Wwox WT* and *KO* groups as determined by immunohistochemical staining on mammary gland tissue sections from 8 week and 12 week virgin female mice (data not shown).

In order to extend the observation period to monitor the effects of *Wwox* ablation in mammary epithelium of the *BK5 KO* model, we performed transplantation experiments of mammary epithelium from 12 week old *BK5 KO* mice into the cleared mammary fat pads of 4–5 week old virgin SCID female mice (n = 10). We followed the SCID recipient mice for 9 additional months and did not observe tumor formation in any of the recipient mice. At the end of the observation period transplanted mammary fat pads were dissected and prepared for whole mounts. The obtained mammary pad whole mounts were evaluated after carmine alum staining confirming the successful grafting of the donor epithelium in 100% of the cases. We did not observe the development of any premalignant or malignant lesions in these whole mount preparations (data not shown).

### Long-term follow up experiments *MMTV-Cre* model

The *MMTV-Cre* line D expresses the Cre recombinase in luminal and myoepithelial cells of the mammary gland and is significantly expressed at approximately 22 days of age [25]. In contrast with the *BK5-Cre KO* model, we observed that *Wwox* deletion by means of the *MMTV-Cre* system had no effect in survival and natural aging. We followed 60 *MMTV-Cre(+); Wwox^flox/flox^*, i.e. *KO* mice (30 females, 30 males) and 23 *MMTV-Cre(−); Wwox^flox/flox^*, i.e. *WT* mice (13 females, 10 males) for up to 1.4 years and observed no statistical difference in survival between the two groups. Importantly, and like with the *BK5-Cre* model, when whole mounts or histological sections of mammary glands were analyzed we found no evidence of hyperplasia, premalignant or malignant lesions (Figure 1F). We also observed no evidence of tumor formation in any other tissue known to be affected by *MMTV-Cre* such as the salivary gland [25].

Given the moderate to mild mosaicism in Wwox expression detected in some *MMTV-Cre KO* mice, we investigated whether Wwox-positive epithelial cells could gain an advantage over the Wwox-negative epithelium and repopulate the mammary gland later in life. To this end we analyzed Wwox expression in the mammary glands of *WT* (n = 8) and *KO* (n = 17) mice when they reached 1.4 years of age at the end of the survival study. We found as expected that all *Wwox WT* mammary glands were still strongly positive for Wwox expression and we observed that over half of the *Wwox KO* glands were completely negative (n = 9) for Wwox expression indicating effective deletion of Wwox while the remaining samples (n = 8) showed some mosaicism with areas displaying some epithelial structures positive and other negative for Wwox expression, indicating that even though the mosaicism remained the Wwox expressing cells do not overtake the mammary gland.

### *Wwox* deletion results in aberrant mammary branching morphogenesis

**Figure 3:**
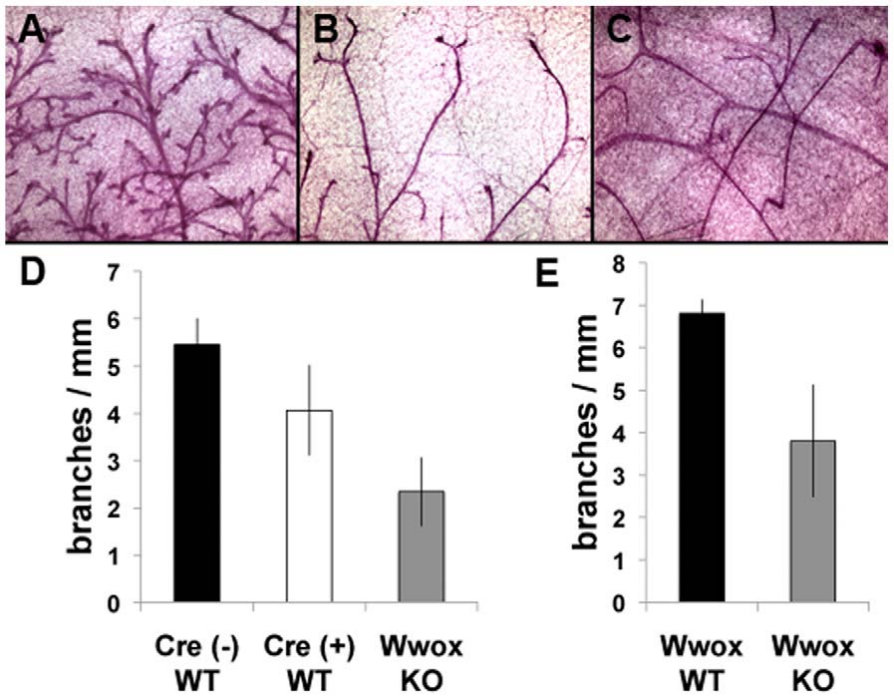
Deletion of *Wwox* results in defective mammary branching and lobuloalveolar development. (A–B) Representative microphotographs obtained from whole mounts of 12 week virgin *BK5 Wwox WT* (A) and *KO* (B) mammary glands. The impaired branching phenotype was also dramatically evident in mammary gland from *MMTV KO* mice as illustrated in (C), extreme case of branching impairment in a 24 week virgin *MMTV Wwox KO* mammary gland. (D) The average number of branches/mm of ductal length was quantified for *BK5-Cre (−); Wwox^flox/flox^* (Cre (−) WT, n = 4), *BK5-Cre (+); Wwox^+/+^* (Cre (+) WT, n = 4), and *BK5-Cre (+); Wwox^flox/flox^* (Wwox KO, n = 5). A small yet statistically insignificant (p = 0.06) decrease in branching can be seen in Cre(+) WT as compared to Cre(−) WT. The decreased branching found in *Wwox KO* mammary glands was significant when compared to either Cre (−) WT (p = 0.0002) or Cre (+) WT (p = 0.018). (E) Quantification of branches/mm ductal length in 24 week virgin *MMTV Wwox WT* controls (*MMTV-Cre (−); Wwox^flox/flox^* and *MMTV-Cre (+); Wwox^+/+^* were grouped together, n = 4) and *MMTV Wwox KO* (*MMTV-Cre(+); Wwox^flox/flox^*, n = 4) mammary glands. Branching was statistically significantly decreased in *Wwox KO* when compared to *WT* controls (p = 0.017). Error bars indicate standard deviation.

By simple microscopic inspection of the mammary whole mounts obtained from the *BK5 Wwox KO* and *MMTV Wwox KO* mice we observed a significant impairment in branching development when comparing with the *WT* counterparts. In some cases the impairment in mammary fat pad ductal invasion and expansion was very dramatic and extreme developing just a rudimentary mammary epithelium or just a few ductal structures with little or no branching (Figures 3 A–C).

In order to quantitate these observations we performed morphometric determinations of mammary branching. To this end whole mounts of the 4^th^ inguinal mammary glands were collected from virgin female mice at 12 weeks of age from *BK5 Cre(−) WT, BK5 Cre(+) WT* and *BK5 KO*. We observed some impairment in branching morphogenesis in the control glands expressing Cre recombinase when compared with the Cre negative counterparts. However we observed a statistical significant decrease in branching in the mammary glands from the *BK5 Wwox KO* mice when compared with either of the controls (Figures 3 A, B, and D).

Importantly, the impaired branching observed in the *BK5 KO* mammary gland did not impede the mammary epithelium to reach functional maturity and differentiation during pregnancy. Furthermore, *BK5 Wwox KO* mice were able to lactate and expressed similar levels of the differentiation markers *Wap* and *β-casein* as determined by qRT-PCR (data not shown).

In the *MMTV-Cre* model of *Wwox* deletion we also observed a similar phenomenon in reduced branching (Figure 3C). However such phenotype was more heterogeneous than with the *BK5-Cre* model. The branching phenotype in *MMTV Wwox KO* mice ranged from very severe (Figure 3 C) to more similar to wild-type. We did not detect statistically significant differences at 12 weeks of age given the large standard deviations observed in *MMTV Wwox KO* mice (data not shown). However, analyses from older *MMTV-Cre* females (24 weeks of age) detected significant differences in branching (6.8+/−0.32 vs. 3.8+/−1.33 branches/mm, p = 0.017, Figures 3 C and E).

### Global gene expression changes in *Wwox* ablated mammary epithelium

**Figure 4:**
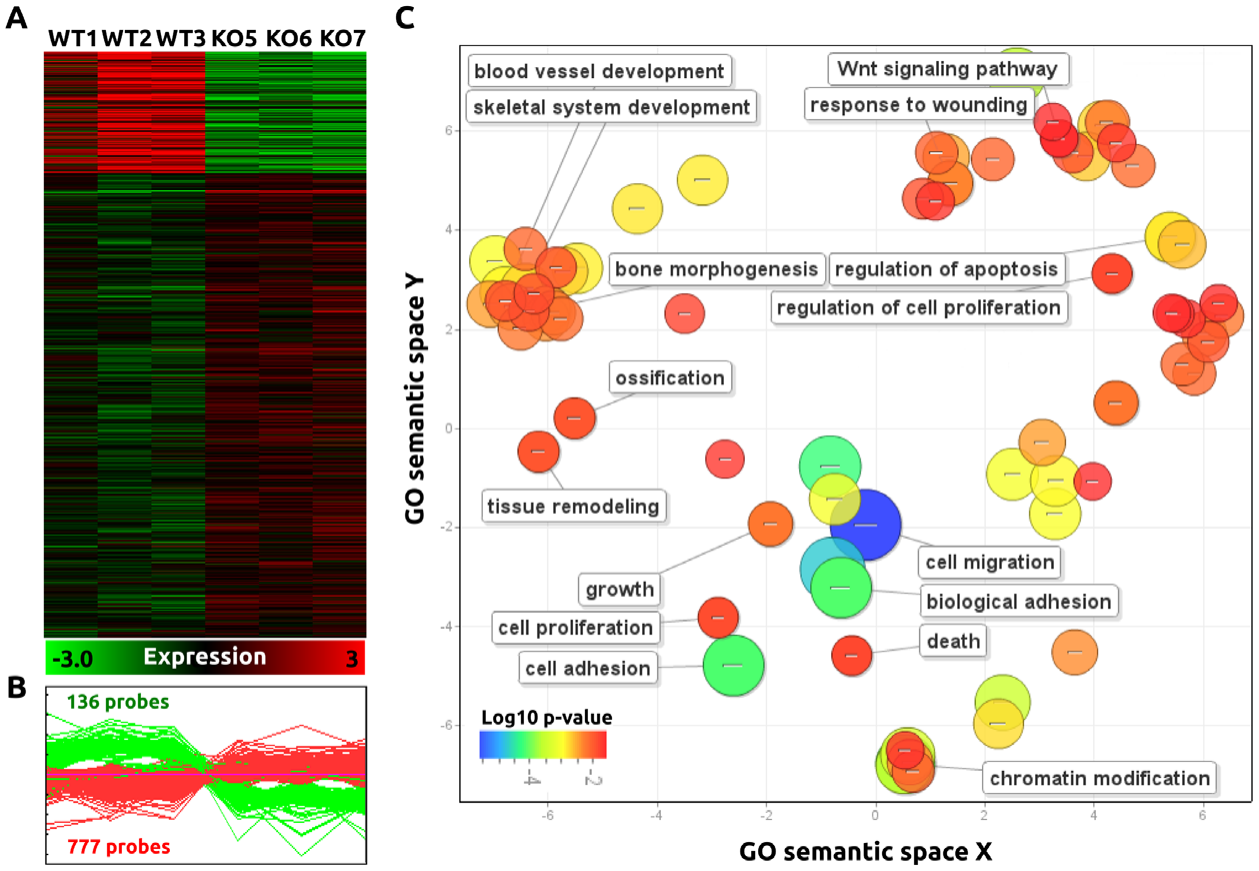
*Wwox-KO BK5-Cre* transgenic mice model gene expression profile study. (A) Heatmap of the differentially expressed genes between *Wwox WT vs. Wwox KO* mammary gland epithelial organoid samples (p<0.01; 2 Fold changes). Color scale at bottom of picture is used to represent expression level: low expression is represented by green, and high expression is represented by red. (B) Expression graph of the 913 deregulated probes (136 probes down-modulated and 777 up-modulated) among *Wwox WT* and *Wwox KO* mammary epithelial organoid samples. (C) Scatterplot graph showing the representative clusters, after redundancy reduction of the statistical significant GO terms (p<0.025) enriched in the deregulated gene list, in a two dimensional space related to GO terms' semantic similarities. Bubble color indicates the p-value of GO terms (expressed as Log10 p-value) and bubble size indicates the frequency of the GO term in the underlying GOA database (bubbles of more general terms are larger).

To determine the effect of *Wwox* ablation on global gene expression in mammary epithelium we performed gene expression microarray analyses on RNA obtained from 8 week old *BK5 Wwox KO* and *WT* mammary epithelium (n = 3 *WT* mice and 3 *KO* mice). We decided to pursue these studies with the *BK5-Cre* model given the uniformity and efficiency in *Wwox* ablation. The statistical analysis of the Affymetrix gene chip expression profiling data identified 913 probes differentially expressed between *Wwox KO* and *Wwox WT* mammary gland epithelium (p<0.01; 2 fold changes). Among the 913 probes, 777 were up-modulated and 136 were down-modulated in the mammary gland epithelium derived from the *Wwox KO* mice model (Figure 4A and B; Table S1). We used the DAVID resource for automated annotation and enrichment analysis of the differentially expressed genes based on GO database. In addition, we employed REVIGO resource for summarization and visualization of the significant GO term semantic similarities (p<0.025). Among the statistically significant over-represented categories under Biological Processes, we found the Wnt signaling pathway, skeletal system development/bone morphogenesis, and tissue remodeling related genes (Figure 4 C). In addition, categories of genes found in cell migration and adhesion were also highly enriched related genes in the *Wwox-KO* mammary gland expression profile.

### Validation of gene expression changes

**Figure 5:**
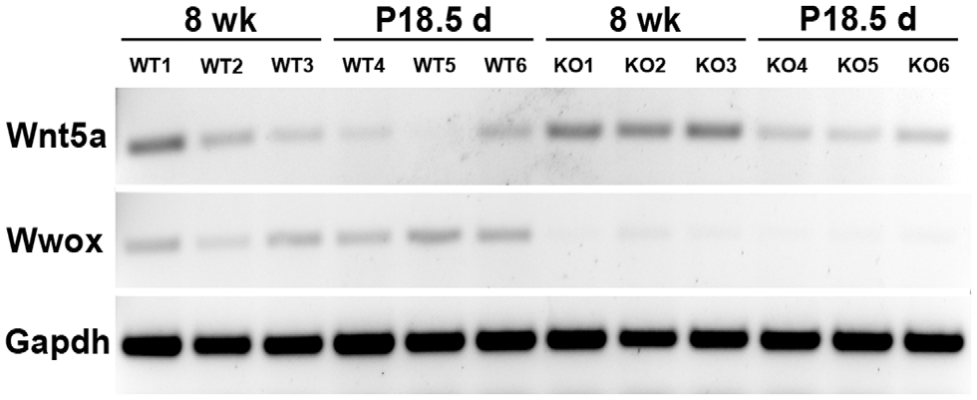
*Wnt5a* expression increases in *BK5 Wwox KO* mammary epithelium. Semi-quantitative RT-PCR was performed on cDNA synthesized from RNA obtained from mammary epithelial organoids (3 different animals per group) as discussed in Materials and Methods. Lanes from left to right are: (lanes 1–3) 8 week virgin *Wwox WT*, (lanes 4–6) P18.5 *Wwox WT*, (lanes 7–9) 8 week virgin *Wwox KO*, and (lanes 10–12) P18.5 *Wwox KO*. *Wwox* expression is shown in the middle panel as further verification of *Wwox* ablation. *Gapdh* expression is shown as a normalization control. Each PCR reaction was performed at 24, 26, 28 and 32 cycles to ensure that reaction was in linear range. Results shown at 24 cycles.

One of the most significantly upregulated genes in *Wwox-KO* mammary epithelium was the non-canonical Wnt pathway ligand *Wnt5a* (fold change = 3.01, p = 0.000057). *Wnt5a* is expressed during all stages of mammary gland development except during lactation [26]. We sought to validate the findings of our microarray by performing semi-quantitative RT-PCR on RNA from the mammary gland epithelial organoids of three 8 week old *WT* mice compared to three *Wwox-KO* mice (Figure 5, lanes 1–3 and 7–9). *Wnt5a* expression was observed to be variable in unsynchronized mice; mammary epithelial samples from two *WT* mice displayed very low levels of Wnt5a expression while one displayed high *Wnt5a* expression. We observed that all *Wwox-KO* mammary epithelial samples exhibited upregulation of *Wnt5a*. We then tested this expression in the pregnancy synchronized epithelium of P18.5 mice and found that 2 out of 3 *WT* mammary glands displayed low levels of *Wnt5a* expression while all *Wwox-KO* mammary glands tested exhibited increased levels of *Wnt5a* mRNA (Figure 5, lanes 4–6 and 10–12).

**Figure 6:**
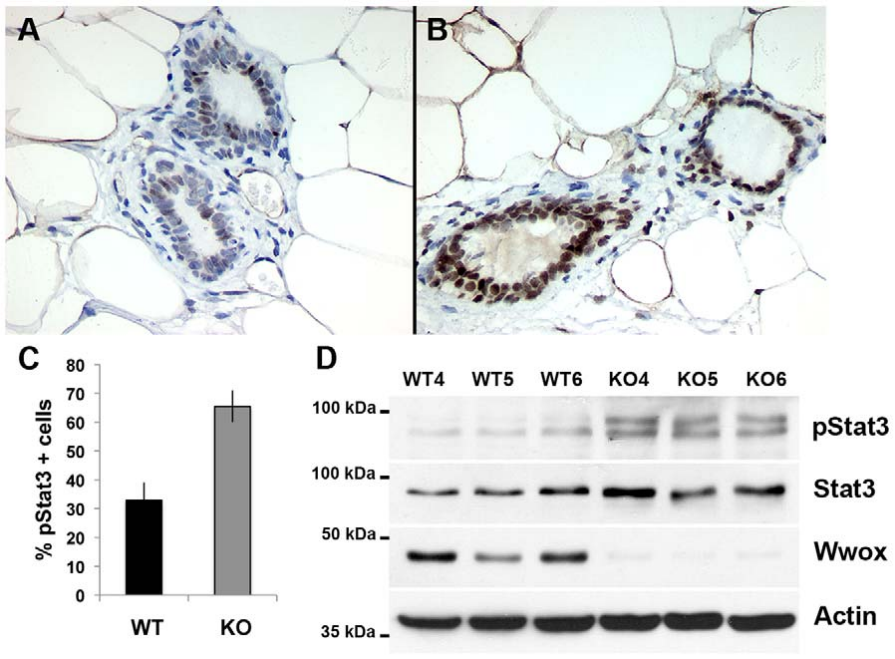
*Wwox* deletion correlates with increased phospho-Stat3 protein in mammary epithelium. (A–B) Immunostaining for phospho-Stat3 (pStat3) protein in histological sections from 12 week virgin *BK5 Wwox WT* (A) and *Wwox KO* (B) mammary glands. Strong nuclear staining can be seen in the majority of cells in *Wwox KO* sections. (C) Quantification of percentage of cells positive for pStat3 staining in *Wwox WT* (n = 3) and *Wwox KO* (n = 3) histological sections. A total of 1000 cells were counted from random fields within each sample. (D) Whole cell lysates from mammary epithelial organoids from P18.5 *BK5 Wwox WT* (n = 3; WT4, WT5, WT6) and *Wwox KO* (n = 3; KO4, KO5, KO6) mice, were probed pStat3 levels. Wwox protein levels are shown to verify Wwox knockdown. Total Stat3 levels are shown as a control and Actin protein levels are shown as a loading control.

We then sought to determine possible mechanisms by which *Wnt5a* expression could be upregulated in *Wwox-KO* mammary gland. IL-6 induced Jak/Stat3 signaling has been shown to activate *Wnt5a* expression in many different cell types [27], [28]. From our microarray analysis we identified significant up-regulation of several genes related with the Jak/Stat3 signaling pathway (see Table S1). For instance, *IL6st*, the coreceptor and signal transducer of the pathway, was upregulated 4.17 fold (p = 0.005), *Jak1* was upregulated 3.04 fold (p = 0.003) and *Stat3* was upregulated 2.4 fold (p = 0.01). To determine whether Stat3 was activated (i.e. phosphorylated) we analyzed phospho-Stat3 expression by means of immunohistochemistry in mammary gland samples from *BK5 Wwox WT* (n = 3) and *KO* (n = 3) mammary glands. We found that *Wwox KO* mammary epithelium had a significantly higher number of cells that were positive for strong nuclear phospho-Stat3 staining (p<0.003) (Figures 6 A–C). We also compared levels of phospho-Stat3 by means of Western blotting in *BK5-Cre WT* and *KO* epithelial organoids at p18.5, we indeed found a significant increase in phospho-Stat3 expression in the KO samples (Figure 6D).

In the canonical Wnt pathway we found that several genes were significantly upregulated in *Wwox KO* mammary epithelium. This pathway was also highlighted by the GO analysis of the gene expression data (Figure 4C). *Gsk3b* was increased 6.65 fold (p = 0.0044). In addition, we identified over-expression of the *Lrp6* coreceptor (1.8 fold; p = 0.0004) and the signal transducer *Dishevelled (Dvl1)* (1.8 fold; p = 0.0033) at a less stringent statistical cutoff (under 2 fold changes). The *β-catenin* gene was upregulated 3.85 fold (p = 0.0007) in our microarray. To determine whether the noted upregulation of *β-catenin* mRNA and other canonical pathway components resulted in an increase of nuclear localized *β-catenin* protein we performed immunohistochemical analysis on mammary gland sections from 12 week old virgin *BK5-Cre* mice. We found that all protein was localized to cytoplasm and cell membrane, no nuclear localization was noted and no differences were observed between *BK5 WT* and *KO* mammary samples, indicating that likely most *β-catenin* is degraded in the cytoplasmic compartment and does not translate in hyperactivation of said pathway. This is consistent with the findings that no classical target genes of the canonical Wnt pathway were observed upregulated in *KO* mammary glands (e.g. *Cyclin D1, Myc, Fgf20*) and no change in proliferation was observed.

## Discussion

Previous mouse *Wwox KO* models have shown that deletion of this gene dramatically affects the lifespan of the animal, all mice die by 3–4 weeks of age thus making observations in adult tissues impossible [18], [19]. Here we present the first report on the effects of conditional *Wwox* deletion in developing and mature mouse mammary epithelium.

One of the primary goals of this study was to determine whether deleting *Wwox* in the mammary gland leads to tumor formation. Importantly, we found no evidence of tumor formation in the mammary gland or any other tissue by either of the two conditional KO approaches utilized, even if these mice were monitored up to 1.4 years of age. This strongly suggests that *Wwox* does not behave as a classical highly penetrant tumor suppressor gene in mammary gland epithelium as previously thought [4]. Our studies clearly indicate that loss of *Wwox* expression is not sufficient for tumor formation. Furthermore, our findings are in clear disagreement with previous speculation suggesting that *Wwox* haploinsufficiency is tumorigenic [18], [29], [30], [31]. Importantly, *Wwox* ablation did not lead to the development of hyperplasia or preneoplastic lesions and no increase in cell proliferation was observed in mammary gland epithelium. This suggests that Wwox loss of expression is perhaps of more relevance during the progression stages of tumorigenesis rather than in the initiation of neoplasia. In fact, we did find that loss of Wwox in the mammary gland is associated with an increase in the expression of several genes associated with tumor progression.

In previous studies, Abdeen et al reported increased susceptibility to mammary carcinogenesis in Wwox+/−mice that were backcrossed onto the mammary tumor-susceptible C3H genetic background. Thus, an effect of Wwox in tumor initiation cannot be ruled out at this point. There is also a possibility that genetic background may play a role in masking some mammary phenotypic effects of Wwox ablation since our studies were performed in a mixed 129SV/C57Bl/6 genetic background. However, it is unlikely that we would not see any signs of mammary cancer predisposition as described throughout this report.

We further observed that *Wwox* ablation results in a significant impairment in mammary gland ductal branching in virgin mice. Perhaps the lack of Wwox expression affects the ability of epithelial cells to invade the mammary fat pad during development. Furthermore several genes associated with tissue remodeling and extracellular matrix interactions were observed deregulated. For instance, *Timp2* and *Timp3* were both found significantly upregulated in *Wwox KO* epithelium (Table S1).

However, the observed defects in mammary gland morphogenesis did not inhibit normal functional differentiation since both *BK5* and *MMTV Wwox KO* mice are able to produce mature mammary epithelial gland structures during pregnancy leading to milk production and normal lactation.

In our gene expression studies we found that the *Wnt5a* gene is significantly upregulated in *Wwox*-ablated mammary epithelium. The Wnt5a ligand primarily signals through the planar cell polarity pathway but has the potential to activate the canonical Wnt signaling pathway as well depending on which set of coreceptors pair with the Frizzled receptor upon Wnt5a binding [32]. High levels of Wnt5a expression have been associated with osteosarcoma [33], prostate carcinoma [34] and metastatic melanoma [35]. Interestingly in normal tissue such as the developing mammary gland high levels of Wnt5a are associated with decreased cell proliferation and delayed development [36]. Furthermore, *Wnt5a* over-expression has been shown to lead to developing a very similar phenotype to what we observed in *Wwox KO* mammary epithelium. *Wnt5a* overexpression inhibits ductal extension and lateral branching while *Wnt5a^−/−^* mammary tissue exhibits an accelerated developmental capacity compared with wild-type tissue [36].

A number of different cell signaling pathways have been shown to activate transcription of the *Wnt5a* gene such as TGFβ, TNFα, Hedgehog, and IL-6/Jak/Stat3 (reviewed in [37]). We chose to focus on the Jak/Stat3 pathway not only because of its association with *Wnt5a* expression but also because several transcripts of said pathway were detected upregulated in *Wwox KO* mammary epithelium.

Mammalian *Wnt5a* is known to be a Stat3-target gene due to tandem Stat-binding sites found in a region of intron 4 of the gene [27]. In the mouse mammary gland, activation of the transcription factor Stat3 by tyrosine phosphorylation has primarily been associated with inducing the transcription of genes involved in the initiation of cell death and tissue remodeling during mammary gland involution post lactation [38]. Previous analyses for phospho-Stat3 have shown that Stat3 is weakly activated in virgin mammary gland, turned off at the onset of pregnancy and reactivated for a short time at the end of pregnancy and the beginning of lactation. Stat3 signaling appears to remain silent for the duration of lactation until it is strongly reactivated at the beginning of involution [39]. In our studies we observed that phospho-Stat3 levels are significantly increased in *Wwox KO* mammary epithelium from virgin and pregnant mice (P18.5). Taking its role in involution into account we would expect to observe increased apoptosis due to the increased activated Stat3 but we saw no significant difference in cell death between *WT* and *Wwox KO* mammary glands (data not shown). In regards to tumorigenesis, despite having a pro-apoptotic function in mammary gland involution, Stat3 has been found to be constitutively active in breast cancers at low levels and these cancers appear to become addicted to Stat3 [39]. Further studies will be required to better understand the interplay between loss of WWOX function and Stat3 activation.

We also observed that the expression of several components of the canonical Wnt pathway were upregulated in *Wwox KO* mammary epithelium. Inhibition of WWOX has previously been associated with increased signaling through this pathway as shown by stabilization of the β-catenin protein as well as increased transcriptional activity in cell lines. It was shown that WWOX binds Dvl-2 and it was proposed that WWOX could play a role in sequestering such signal transducer in the cytoplasmic compartment [40]. Even though we observed an increase in the expression of *β-catenin* at gene expression level, we did not detect active β-catenin nuclear translocation nor did we observe an increase in the expression of known transcriptional target genes such as *Cyclin D1, Myc or Fgf20* or increase in cell proliferation. Further experiments will be needed to clarify the effect of *Wwox* ablation on the canonical Wnt signaling pathway.

In summary, we report the first characterization of tissue specific targeted ablation of the putative tumor suppressor gene *Wwox* in adult mouse tissues. We demonstrated that conditional deletion of Wwox in the mouse mammary gland does not result in tumorigenicity. The fact that WWOX expression is lost in multiple neoplasias by a variety of mechanisms is undeniable and very well documented [4], [5], [7], [41]. Thus, our data indicate that WWOX does not behave as a classical highly penetrant tumor suppressor gene and more likely the loss of WWOX expression is related to tumor progression. Experiments are in progress to determine whether *Wwox* ablation plays a role in mammary gland tumor progression.

## Materials and Methods

### Ethics statement

Mice used in these studies were maintained in a clean, modified-barrier animal facility, fed regular commercial mouse diet (Harlan Lab, Indianapolis, IN) under controlled light (12L∶12D) and temperature (68–74°F). All animal research was conducted in facilities accredited by the Association for Assessment and Accreditation of Laboratory Animal Care International at the University of Texas, M.D. Anderson Cancer Center, Science Park, following international guidelines and all research was specifically approved by the University of Texas M. D. Anderson Cancer Center Institutional Animal Care and Use Committee, (Animal Welfare Assurance Number A3343-01).

### Generation of conditional *Wwox* knockout mice using *BK5-Cre* and *MMTV-Cre* mediated deletion

Female *Wwox^flox/flox^* mice (129SV/C57Bl/6 background) were bred with male mice homozygous for the *Cre* recombinase gene under control of either the *bovine keratin 5* (*BK5*) promoter or the *MMTV* promoter (Line D, [25]) to generate the *Wwox^+/ΔCre^* F1 generation. Generation of *Wwox^flox/flox^* mice is discussed elsewhere [19]. *BK5-Cre* transgenic mice (129SV/C57Bl/6 background) were obtained from the laboratory of Dr. David Johnson (MDACC, Science Park) while the *MMTV-Cre* transgenic mice (129SV/C57Bl/6 background) were obtained from Jackson Labs (stock #003553) Genotypes were determined by PCR using the oligonucleotide primers: Cre F: 5′-GCCTGCATTACCGGTCGATGCAACG-3′ Cre R: 5′-GTGGCAGATGGCGCGGCAACACCAT-3′ Wwox-N1: 5′-ATGGGCCGAAACTGGAGCTCAGAA-3′ Wwox-N2: 5′-TCAGCAACTCACTCTGGCTTCAAC-3′ Wwox-L: 5′-GCATACATTATACGAAGTTATTCGAG-3′.

### Mammary gland whole mount preparation, histology and morphometric analysis

For whole mounts, the 4^th^ inguinal mammary glands were dissected, spread onto glass slides and fixed with formalin overnight and stained with carmine alum by standard procedures. Mammary gland whole mount slides were then scanned for image analysis and morphometry using the ScanScope CS system (Aperio Technologies, Inc., Vista, CA) and scans were analyzed for morphometric features such as number of branches per mm of ductal length.

### Immunohistochemistry

For immunohistochemistry, formalin-fixed paraffin sections from mammary glands were deparaffinized and tissue sections were treated with 10 mM Citrate buffer (pH 6.0; 15 min, microwave) for antigen unmasking and Biocare blocking reagent (Biocare) for blocking endogenous immunoglobulin in cases using primary antibodies. Staining was then performed using DAB (Dako) according to manufacturer's instructions. Primary antibodies used were rabbit anti-Wwox [14] (1∶50 dilution), rabbit anti-pSTAT3 (Cell Signaling Technology, 1∶50 dilution), rabbit anti-Estrogen Receptor alpha (Santa Cruz, 1∶500 dilution), mouse anti-Progesterone Receptor (Abcam, 1∶100 dilution) and rabbit anti-Ki67 (Bethyl, 1∶250 dilution). Tissue sections were counterstained with hematoxylin. Quantification of cells that were positive for pSTAT3, ERα, PR or Ki67 was performed by scanning each stained section with the ScanScope system as described above. A thousand epithelial cells from random fields within the tissue section were counted to determine the percentage of cells that were positive for the respective staining.

### Mammary gland transplantation experiments

Twelve week old virgin BK5-Cre Wwox KO mice served as mammary epithelium donors. Mammary tissues were minced into small pieces (∼1 mm^3^) and were used for transplantation into recipient mice. Recipient mice (n = 10) were 4 week old female SCID mice whose left and right 4^th^ mammary fat pads (MFPs) had been cleared as previously described [42]. The sites of the left MFPs were inoculated with mammary epithelial cells harvested from *BK5-Cre Wwox*-*KO* mice. Recipient mice were monitored for 10 months and transplanted MFPs were dissected. Analysis of MFPs was done by whole mount and histological examinations.

### Microarray data processing, statistical and data mining analyses

Mammary gland epithelial organoids were obtained as previously described [43] from three *WWOX*-*KO* mice (*BK5-cre+; WWOX ^flox/flox^*) and from three *WWOX-WT* mice (*BK5-cre -; WWOX ^flox/flox^*). Total RNA was isolated and purified using the TRIzol/RNeasy Kit (Qiagen) from each mammary gland sample. RNA concentration and integrity were measured on an Agilent Bioanalyzer RNA 6000 Nanochip (Agilent Technologies). Five micrograms of total RNA per sample were used for cDNA synthesis, labeling and hybridization to whole genome Affymetrix GeneChip Mouse Genome 430 2.0 Arrays. Labeling and hybridization to Affymetrix GeneChips was carried out at the Genomics Core Facility at the MDACC. Briefly, we carried out QC and normalization procedures in R/Bioconductor [44] using the simpleaffy package [45].

To identify differentially expressed genes between the WWOX-WT vs. WWOX-KO mammary gland organoid expression profiles, we utilized the modified two-sample t-test using Limma package [46]. Raw datasets have been submitted to NCBI GEO database with accession number: GSE36665. Heatmap visualization of differentially expressed transcripts were done with the MultiExperiment Viewer software (MeV 4.8) [47]. For automated functional annotation and gene enrichment analysis, we used the Database for Annotation, Visualization and Integrated Discovery (DAVID) [48]. The DAVID resource calculates over-representation of specific biological themes/pathways with respect to the total number of genes assayed and annotated. REViGO resource was employed to summarize and visualize the enriched GO terms in a scatterplot graph based on the p-values obtained by DAVID. This allows the identification of biological themes/pathways within a specific list of differentially expressed genes.

### RNA isolation and real time and semi-quantitative RT-PCR

Total RNA was isolated from mammary epithelial organoids from *Wwox* WT and KO female mice (n = 3 different mice in each group) using Trizol (Life Technologies) extraction and then cleaned up with Qiagen RNeasy Mini Kit; 300 ng of total RNA was used for reverse transcription using the High Capacity cDNA Reverse Transcription kit (Applied Biosystems). One uL of the 20 uL reverse transcription reaction was used for real-time PCR ran on the 7900HT real-time PCR system (Applied Biosystems). Taqman Gene Expression Assays (Applied Biosystems) for mouse *Wwox* (assay ID: Mm01247380_m1) and 18S rRNA (assay ID: Hs99999901_s1) were used in separate reactions to determine *Wwox* expression relative to 18S expression in each sample. Experiments were performed in triplicate. Wwox expression was normalized first to 18S expression within each sample and then to WT levels. For semi-quantitative RT-PCR 1 uL of cDNA reaction was added to a PCR reaction using the GoTaq Green Master Mix (Promega). Forward and reverse primers for mouse *Wnt5a*, *Wwox* and *Gapdh* were added to final concentration of 2 pg/uL and are as follows: *Wnt5a* F 5′-TCCTTCGCCCAGGTTGTTAT-3′, *Wnt5a* R 5′-GCAGAGAGGCTGTGCACCTA-3′, *Wwox* F 5′-TGGAGCAAATTCGGGAATAG-3′, *Wwox* R 5′-ATTGCTTCCACCTTGGCTTT-3′, *Gapdh* F 5′-CGTCCCGTAGACAAAATGGT-3′, *Gapdh* R 5′-TCAATGAAGGGGTCGTTGAT-3′. Reactions were run on a BioRad iCycler under standard conditions for 24, 28 and 32 cycles to ensure that reaction conditions were in linear range. PCR reaction products were electrophoresed on 2% agarose gel, stained with GelRed Nucleic Acid Stain (Phenix) and visualized under UV.

### Western blot analysis

To monitor endogenous activation of the Stat pathway in mammary epithelium total protein extracts were prepared from the 4^th^ inguinal mammary epithelial organoids. Protein isolation was done using RIPA buffer (50 mM Tris buffer pH 7.5, 150 mM NaCl, 0.25% sodium deoxycholate, 10 mM EDTA, 1% NP40) with protease inhibitor cocktail (Roche, Mannheim, Germany) and the phosphatase inhibitors sodium vanadate (2 mM) and sodium fluoride (5 mM). For western blotting, 20 ug of total protein was separated on 12.5% SDS-PAGE and transferred to a PVDF membrane. Membranes were blocked and antibodies were diluted in 5% dry milk/TBST. Primary antibodies used were: rabbit anti-Wwox (1∶2000) [7], rabbit anti-phospho-Stat3 (Santa Cruz, 1∶1000) and mouse anti-Stat3 (Santa Cruz, 1∶1000). HRP conjugated secondary antibodies were used followed by chemiluminescence autoradiography. Actin was used as the protein loading control and it was detected using monoclonal anti-actin antibody (Sigma, 1∶1000) and HRP conjugated anti-mouse secondary antibody (1∶5000). Immunodetection was performed using ECL Western Blotting Detection Reagents (GE Healthcare) as described by the manufacturer.

## Supporting Information

Figure S1 **Conditional BK5 Wwox KO mice die prematurely; Kaplan-Meier survival curve of mice with BK5-Cre mediated Wwox ablation.**
(DOCX)

Figure S2 **Cytokeratin 5 immunostaining in** ***BK5 KO*** **mammary gland (10 wk old virgin mouse).** As can be observed, K5 staining is limited to the basal layer of every epithelial structure as in normal wild type mammary gland. No obvious abnormalities in epithelial differentiation were detected.
(DOCX)

Table S1 List of 913 probes from Affymetrix gene expression profile differentially expressed between *Wwox KO* and *Wwox WT* mammary gland epithelium (p<0.01; 2 fold changes).
(XLS)
