# Supplementary material for: Conditional Wwox Deletion in Mouse Mammary Gland by Means of Two Cre Recombinase Approaches
Source: PLoS One. 2012 May 4;7(5):e36618. doi: 10.1371/journal.pone.0036618 (PMC3344920; doi:10.1371/journal.pone.0036618)

Figure S1. Conditional BK5 Wwox KO mice die prematurely; Kaplan-Meier survival curve of mice with BK5-Cre mediated Wwox ablation.


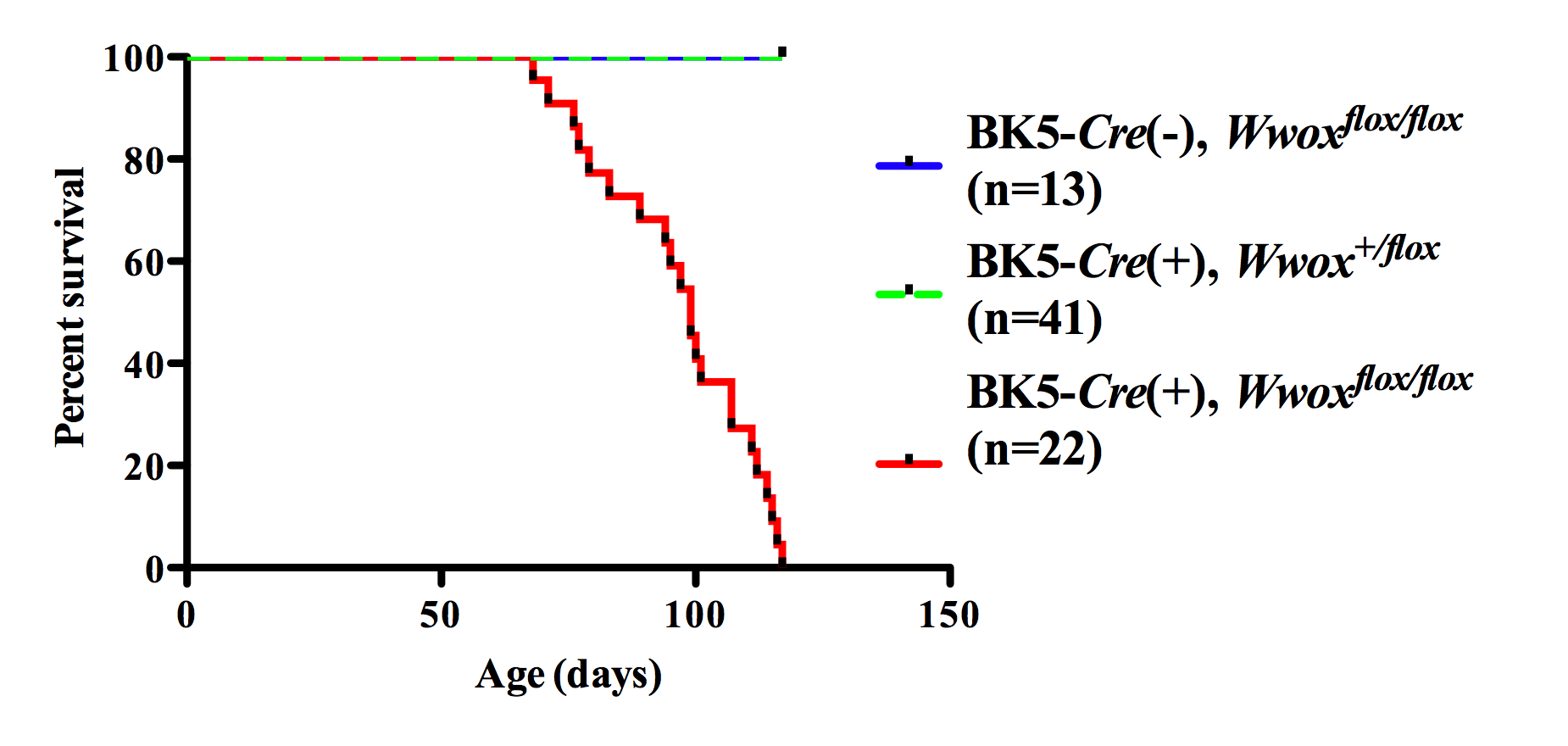

Supplement: Figure S1 — Conditional BK5 Wwox KO mice die prematurely; Kaplan-Meier survival curve of mice with BK5-Cre mediated Wwox ablation. (DOCX) [file pone.0036618.s001.docx]
