# Supplementary material for: Conditional Wwox Deletion in Mouse Mammary Gland by Means of Two Cre Recombinase Approaches
Source: PLoS One. 2012 May 4;7(5):e36618. doi: 10.1371/journal.pone.0036618 (PMC3344920; doi:10.1371/journal.pone.0036618)
Supplement: Figure S2 — Cytokeratin 5 immunostaining in BK5 KO mammary gland (10 wk old virgin mouse). As can be observed, K5 staining is limited to the basal layer of every epithelial structure as in normal wild type mammary gland. No obvious abnormalities in epithelial differentiation were detected. (DOCX) [file pone.0036618.s002.docx]

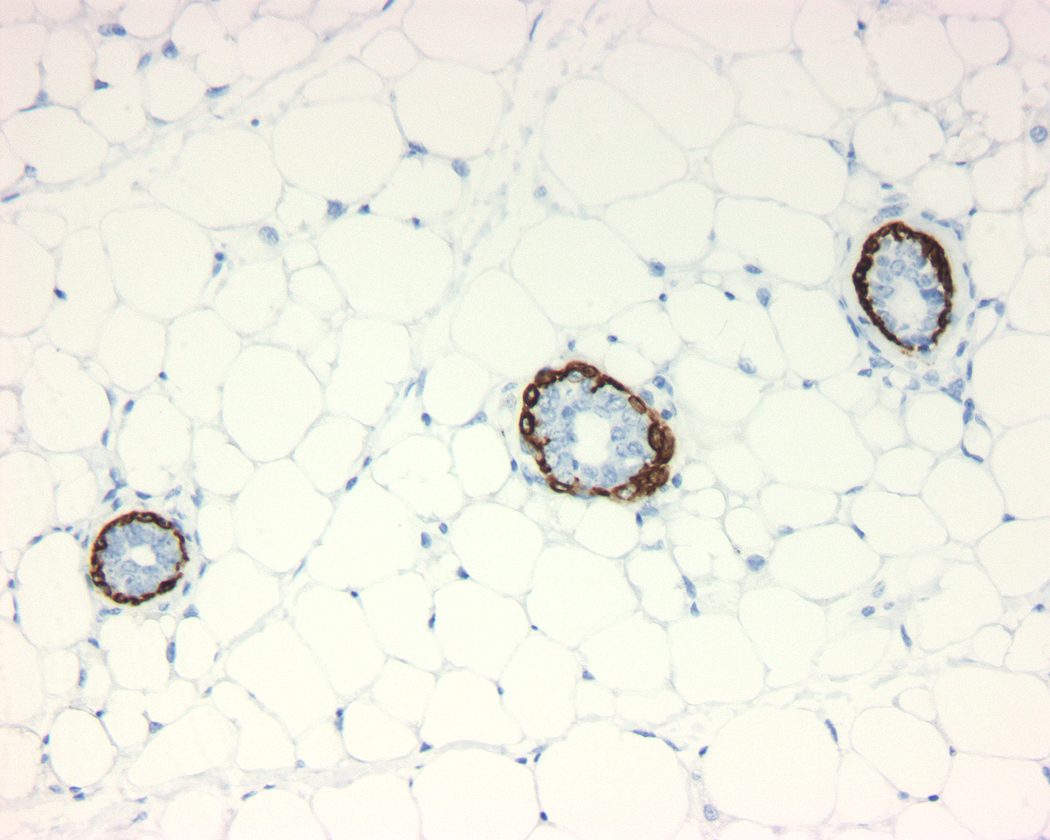


**Figure S2:** Cytokeratin 5 immunostaining in *BK5 KO* mammary gland (10 wk old virgin mouse). As can be observed, K5 staining is limited to the basal layer of every epithelial structure as in normal wild type mammary gland. No obvious abnormalities in epithelial differentiation were detected.
